# Effect of Menopausal Hormone Therapy on the Vaginal Microbiota and Genitourinary Syndrome of Menopause in Chinese Menopausal Women

**DOI:** 10.3389/fmicb.2020.590877

**Published:** 2020-11-20

**Authors:** Lulu Geng, Wenjun Huang, Susu Jiang, Yanwei Zheng, Yibei Zhou, Yang Zhou, Jiangshan Hu, Ping Li, Minfang Tao

**Affiliations:** Department of Gynaecology and Obstetrics, Shanghai Jiao Tong University Affiliated Sixth People’s Hospital, Shanghai, China

**Keywords:** menopause, vaginal microbiota, genitourinary syndrome of menopause, menopausal hormone therapy, 16S rRNA gene sequencing, tibolone

## Abstract

Genitourinary syndrome of menopause (GSM) is a chronic and progressive condition with a series of vulvovaginal, sexual, and lower urinary tract discomforts, mainly due to hypoestrogenism. Menopausal hormone therapy (MHT) has generally been considered as the most effective treatment for GSM. In addition, vaginal microbiota is of particular significance to gynecological and reproductive illnesses and potentially has some intimate connections with GSM. Consequently, we sought to evaluate how MHT impacts the composition and structure of vaginal microbiota while alleviating GSM in Chinese menopausal women aged 45–65 years, which has not been investigated previously. 16S rRNA gene sequencing was performed to analyze microbial diversity and composition using vaginal swabs obtained from 100 menopausal women, classified as MHT women who have been taking tibolone regularly (*n* = 50) and non-treated women who never received any treatment (*n* = 50). Vaginal Health Index Score (VHIS) and GSM symptoms inquiry were also performed. We found that the vaginal microbial diversity decreased and that the abundance of *Lactobacillus* increased to be the dominant proportion significantly in the MHT group, in considerable contrast to vaginal microbiota of the non-treated group, which significantly comprised several anaerobic bacteria, namely, *Gardnerella*, *Prevotella*, *Escherichia-Shigella*, *Streptococcus*, *Atopobium*, *Aerococcus*, *Anaerotruncus*, and *Anaerococcus*. In this study, women without any MHT had significantly more severe GSM symptoms than those receiving tibolone, especially with regard to vulvovaginal dryness and burning, as well as decreased libido (*P* < 0.01). However, there was no significant difference in the severity of urological symptoms between the groups (*P* > 0.05). Furthermore, *Lactobacillus* was demonstrated to be associated with VHIS positively (*r* = 0.626, *P* < 0.001) and with GSM negatively (*r* = −0.347, *P* < 0.001). We also identified *Chlamydia* (*r* = 0.277, *P* < 0.01) and *Streptococcus* (*r* = 0.270, *P* < 0.01) as having a prominent association with more serious GSM symptoms. Our study provided an elucidation that MHT could notably alleviate GSM and conspicuously reshape the composition of the vaginal microbiota, which is of extreme importance to clinical practice for the management of GSM.

## Introduction

Genitourinary syndrome of menopause (GSM), previously known as vulvovaginal atrophy or atrophic vaginitis, is reported in approximately half of perimenopausal and postmenopausal women worldwide (Portman and Gass, 2014; Geng et al., 2018) and characterized by bothersome disorders interfering with quality of life. It is a collection of symptoms and signs associated with low circulating estrogen levels, commonly including vaginal dryness; dyspareunia; frequent, urgent, and painful urination; recurrent urinary tract infection; incontinence; etc. (Portman and Gass, 2014). However, GSM has not received enough attention from health care providers and patients, causing a delay in management. It is reported that only 25% of patients in Asia were willing to seek medical assistance, and only 24% of them had access to treatment (Chua et al., 2017). Menopausal hormone therapy (MHT) previously has been shown to be the most effective management for GSM (Gandhi et al., 2016; Faubion et al., 2017). As estrogen receptors distribute in diverse tissues and organs of our body, women in the menopausal stage tend to manifest various types of symptoms requiring treatment due to estrogen deficiency. We found that systemic hormone replacement therapy is suitable for a great many cases in our practical clinical work. The human microbiota inhabiting the vagina alter dynamically across different reproductive stages (Gajer et al., 2012). After menopause, endocrine level and vaginal environment change gradually accompanied by changes in vaginal microbial community structure. In general, vaginal dominant colonization with *Lactobacillus* is infrequent compared to premenopausal women (Muhleisen and Herbst-Kralovetz, 2016; Kaur et al., 2020). However, the correlation between menopausal related genitourinary symptoms and vaginal flora is still full of ambiguity. Some studies reported that women with examination findings of atrophy had lower proportion of *Lactobacillus* (Shen et al., 2016; Brotman et al., 2018), whereas other studies did not show this significant association (Mitchell et al., 2017, 2018). While there is evidence of associations between different types of hormone therapy and increased detection of *Lactobacillus* in vaginal environment (Devillard et al., 2004; Mitchell et al., 2018; Gliniewicz et al., 2019), relatively little is known about effects of MHT on the overall vaginal microbial composition and symptoms and signs of GSM, especially in Chinese women.

In this cross-sectional study, we sought to explore vaginal microbial structure and severity of GSM in women who received regular and uniform MHT in comparison with those who did not by using 16S rRNA gene amplicon sequencing to identify microbial taxa. Moreover, we evaluated the severity of common symptoms in GSM, confirmed a transformation in abundance and diversity of vaginal bacteria after MHT, and elucidated particular associations among vaginal microbiota, MHT, and clinical characteristics.

## Materials and Methods

### Subjects’ Data and Sample Collection

Menopausal women were recruited and selected from patients attending the menopause clinic in the Shanghai Jiao Tong University Affiliated Sixth People’s Hospital in China, from February 2017 to December 2018. These participants were divided into two groups of 50 subjects each, according to whether they have received regular MHT (tibolone, a synthetic steroid, 2.5 mg orally per day, was selected as the uniform treatment in this study) at least in the past year. Women were included in the study if they were 45–65 years old and did not have menstruation in the past 60 days. In addition, women were excluded if they had any contraindications of MHT; irregular menstrual bleeding due to disease; induced menopause caused by drug, operation, or chemoradiotherapy; a history of diabetes or severe chronic diseases; or they were pregnant or lactating. Moreover, women receiving intravaginal operation such as gynecological examination within the past 24 h, systemic or vaginal topical antibiotic treatment in the past 30 days, or those with no sexual partner were also excluded from this study. All study subjects signed an informed consent form before participation.

Participants responded to one-on-one questionnaires conducted by trained investigators. Demographic characteristics were first assessed by a questionnaire including age, height, weight, reproductive history, menstruation, employment status, economic level, and years of education. Height and weight were measured to calculate body mass index (BMI). In addition, serum estradiol (E_2_) and follicular stimulating hormone (FSH) levels were abstracted from medical records archived at the Shanghai Jiao Tong University Affiliated Sixth People’s Hospital with participants’ permission. Common menopausal genitourinary symptoms, such as (1) vulvovaginal symptoms including dryness, itching, and burning; (2) urological symptoms including frequent, urgent, and painful urination; recurrent urinary tract infection; and incontinence; and (3) sexual symptom including only decreased libido, were also evaluated by asking women if they had bothersome symptoms described above on a five-point scale ranging from 0 to 4, corresponding to “asymptomatic,” “mild,” “moderate,” “severe,” and “extremely severe,” respectively. Objectively, the severity of GSM was represented by the sum of scores for all seven symptoms.

Then, women underwent a gynecological examination by an experienced gynecologist, during which the Vaginal Health Index Score (VHIS) was measured. The VHIS evaluates the condition of vaginal mucosa through elasticity, vaginal discharge, pH, mucosal integrity, and moisture. A lower VHIS means a worse vaginal physical condition, and if the score is less than 15, it indicates the status of vaginal atrophy (Arêas et al., 2019). Also during this visit, two sterile swabs were used to obtain vaginal secretions samples from the posterior vaginal fornix while avoiding touching the cervix and vulva to avert contamination. One swab was used to detect vaginal pH by a pH test strip (Merck, Darmstadt, Germany) from 4.0 to 7.0 (Huang et al., 2010), and the other was immediately placed in a cryopreservation tube and stored in a −80°C freezer until processed.

### DNA Extraction, Polymerase Chain Reaction Amplification, and Sequencing

Before the DNA extraction, frozen swabs containing vaginal secretions were thawed on the ice in a decontaminated environment. The bacterial genomic DNA was isolated from each specimen using the FastDNA^®^ SPIN Kit for Soil (MP Biomedicals, Irvine, CA, United States), according to the standard manufacturer’s protocol, and stored at −20°C prior to subsequent detection. DNA concentrations and purities were quantified by the NanoDrop^®^ ND-2000 UV-Vis spectrophotometer (Thermo Fisher Scientific, Wilmington, DE, United States), and DNA quality was verified by 1% agarose gel electrophoresis.

The V3–V4 hypervariable region of the bacterial 16S ribosomal RNA gene was amplified using primers (Ling et al., 2017) 338F (5′-ACTCCTACGGGAGGCAGCA-3′) and 806R (5′-GACTACHVGGGTWTCTAAT-3′) under the following thermal-cycling parameters: an initial temperature of 95°C for 3 min, followed by 28 cycles of 95°C for 30 s, 55°C for 30 s, and 72°C for 45 s, and a final extension at 72°C for 10 min. The polymerase chain reaction (PCR) reactions were performed in a mixture in volume of 20 μL, containing 4 μL of 5 × FastPfu Buffer, 2 μL of 2.5 mM dNTPs, 0.8 μL of forward/reverse primer (5 μM), 0.4 μL of FastPfu Polymerase, 10 ng of template DNA, and 10 μL of ddH_2_O. The PCR products were checked on a 2% agarose gel, extracted from the gel, and further purified using the AxyPrep DNA Gel Extraction Kit (Axygen Biosciences, Union City, CA, United States) and then quantified using QuantiFluor^TM^-ST (Promega, Madison, WI, United States). Subsequently, purified amplicons were pooled in equal amounts and were sent for paired-end sequencing (2 ^∗^ 300) on an Illumina MiSeq platform.

### Bioinformatics and Statistical Analysis

Bioinformatics analysis was conducted in QIIME (v 1.9.0) or using R project (v 3.6.3) basically. Raw 16S rRNA gene sequences were analyzed using the Quantitative Insights into Microbial Ecology (QIIME) pipeline (v 1.9.0) as previously described (Caporaso et al., 2010). Briefly, raw sequences were assigned to respective samples based on the sample specific barcode sequences and trimmed through removal of both barcode and primer sequences for further quality filtering. Criteria used to filter the low-quality sequence reads were mainly as follows: sequences with a length less than 150 bp, sequences with an average quality score less than 20, sequences with mononucleotide repeats greater than 8, and sequences containing one or more ambiguous base (Huang et al., 2018). Retained high-quality sequences were clustered into distinct operational taxonomic units (OTUs) at a 97% similarity threshold using QIIME’s UCLUST program (Edgar, 2010), and chimeric sequences were detected and removed with ChimeraSlayer implemented in QIIME (Haas et al., 2011). Finally, representative sequences were aligned against the SILVA database (Quast et al., 2013)^1^ using the Ribosomal Database Project (RDP)^2^ Bayesian classifier that was retrained to assign taxonomy to each OTU at a 70% confidence threshold. Vaginal microbial taxa were confirmed at the phylum, class, order, family, and genus levels in this portion of the study analysis. In order to eliminate the difference of sequencing depth among samples and to minimize the sequencing errors caused by different library sizes, an average and uniform OTUs table was generated by subsampling under the minimum sequencing depth for downstream analysis.

Based on the OTU information, alpha and beta diversities were utilized to examine vaginal microflora structure. Alpha diversity (de la Cuesta-Zuluaga et al., 2019) (considered as “within” sample diversity) indices were calculated using the vegan package in R (v 3.6.3), including Shannon diversity index, Ace richness index, Chao estimator, Simpson diversity index, and Good’s coverage. Beta diversity (Chu et al., 2017) (known as “between” sample diversity) was analyzed using the Bray–Curtis and weighted UniFrac distance matrices. Then the principal coordinate analysis (PCoA) was plotted using R package ggplot with significant clustering in the intergroup Adonis test. The hierarchically clustered heatmap was generated to visualize microbial taxonomy composition and abundance by the R package pheatmap. Briefly, some relevant tables reflecting community structures were generated to reinforce argument explicitly. In addition, the linear discriminant analysis (LDA) effect size (LEfSe) (Segata et al., 2011) modeling was applied for the identification of significantly differential genera between groups based on relative abundance performed in the online Galaxy tools^3^. To define multivariate correlations of clinical characteristics and microbial community structure, Pearson correlation coefficient between them was calculated, and canonical correlation analysis (CCA) (Guo et al., 2016) was implemented in the R package vegan.

Further statistical analysis was performed using SPSS software (v 22.0). For continuous variables, data with a normal distribution were expressed as mean ± standard deviation; otherwise, data were shown as median (lower quartile, upper quartile). For categorical variables, the percentage was used to describe information. To judge differences between groups, independent-samples *t*-test (normal distribution and homogeneity of variance), Mann-Whitney *U*-test (non-normal distribution), χ^2^-test, or Fisher exact test (comparison of rate) was applied. Differences were considered statistically significant with *P* < 0.05.

## Results

### Study Cohorts and the Severity of GSM Symptoms

A total of 100 vaginal samples from 100 menopausal women were available for analysis in this study and classified into two groups: non-treated women without any MHT (*n* = 50) and women on MHT over the past year (*n* = 50, tibolone was selected as the unified medication regimen for MHT in our study). Table 1 describes the main demographic characteristics of each group, and no significant difference was observed between groups except for years since menopause that was longer in women with MHT than in non-treated women.

**TABLE 1:** Demographic characteristics of participants.

Demographics		Non-treated group	MHT group	*P*-values
		**(*n* = 50)**	**(*n* = 50)**	
Age (y, mean ± SD)		53.34 ± 3.33	54.76 ± 4.27	0.067^*a*^
BMI (kg/m^2^, mean ± SD)		22.50 ± 3.54	21.85 ± 2.50	0.291^*a*^
E_2_ [pg/mL, median (Q1–Q3)]		26.5 (17.00–33.25)	28.5 (17.75–37.75)	0.909^*b*^
FSH [mIU/mL, median (Q1–Q3)]		68.16 (51.29–80.08)	59.14 (47.52–68.29)	0.107^*b*^
No. of parity (*n*, %)	≤ 1	43 (86.0%)	43 (86.0%)	1.000^*c*^
	≥ 2	7 (14.0%)	7 (14.0%)	
No. of abortion (*n*, %)	≤ 1	29 (58.0%)	31 (62.0%)	0.683^*c*^
	≥ 2	21 (42.0%)	19 (38.0%)	
Occupational status (*n*, %)	In work	23 (46.0%)	21 (42.0%)	0.687^*c*^
	Retired/unemployed	27 (54.0%)	29 (58.0%)	
Educational length (y, *n*, %)	≤ 6	2 (4.0%)	1 (2.0%)	0.638^*d*^
	6–9	9 (18.0%)	12 (24.0%)	
	9–12	19 (38.0%)	22 (44.0%)	
	> 12	20 (40.0%)	15 (30.0%)	
Economic state (monthly income, yuan, *n*, %)	≤ 3,000	17 (34.0%)	21 (42.0%)	0.533^*c*^
	3,000–5,000	15 (30.0%)	16 (32.0%)	
	> 5,000	18 (36.0%)	13 (26.0%)	

*BMI, body mass index; E_2_, estradiol; FSH, follicular stimulating hormone. ^a^Independent-samples t-test. ^b^Mann-Whitney U-test. ^c^Pearson χ^2^-test. ^d^Fisher exact test.*

**FIGURE 1:**
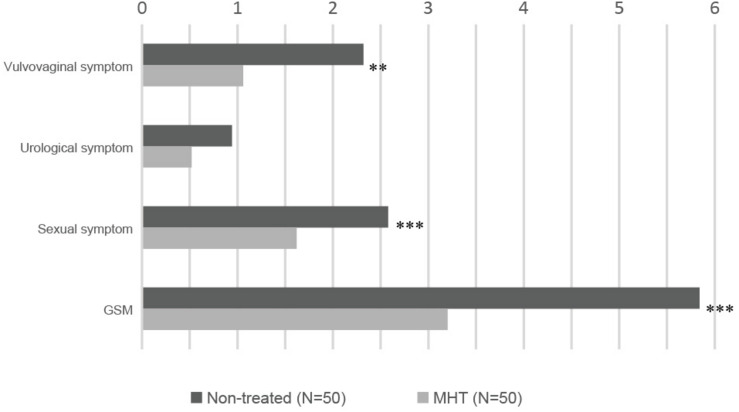
Severity of GSM main symptoms in each group: non-treated vs. MHT. Higher score means more severe symptom; statistical differences are shown in this figure: ^∗∗^*P* < 0.01, ^∗∗∗^*P* < 0.005.

Table 2 shows common symptoms of GSM by severity, from asymptomatic to extremely severe, and Figure 1 demonstrates intuitively the mean score for main symptoms by group. Scores of vaginal symptoms and sexual symptom, as well as GSM, were higher in the non-treated group, indicating that a majority of GSM symptoms were significantly relieved in women treated with MHT (*P* < 0.01). However, there was no significant drop in the severity scores of urological symptoms between the groups (*P* > 0.05).

**TABLE 2:** Common symptoms of GSM.

Common symptoms	Non-treated group (*n* = 50)						MHT group (*n* = 50)						*P*-values
	Asymptomatic	Mild	Moderate	Severe	Extremely severe	Mean ± SD (Mean)	Asymptomatic	Mild	Moderate	Severe	Extremely severe	Mean ± SD (Mean)	
Vulvovaginal symptom						2.32 ± 0.36 (2)						1.06 ± 0.18 (1)	**< 0.01**
Dryness	30.00%	36.00%	20.00%	10.00%	4.00%	1.22 ± 0.16 (1)	58.00%	26.00%	16.00%	0.00%	0.00%	0.58 ± 0.11 (0)	**<0.005**
Itching	62.00%	20.00%	6.00%	12.00%	0.00%	0.68 ± 0.15 (0)	68.00%	22.00%	10.00%	0.00%	0.00%	0.42 ± 0.10 (0)	0.366
Burning	78.00%	10.00%	4.00%	8.00%	0.00%	0.42 ± 0.13 (0)	96.00%	2.00%	2.00%	0.00%	0.00%	0.06 ± 0.04 (0)	**< 0.01**
Urological symptom						0.94 ± 0.19 (0)						0.52 ± 0.16 (0)	0.051
Frequent, urgent, and painful urination	70.00%	18.00%	10.00%	2.00%	0.00%	0.44 ± 0.11 (0)	84.00%	14.00%	2.00%	0.00%	0.00%	0.18 ± 0.06 (0)	0.072
Recurrent urinary tract infection	90.00%	2.00%	8.00%	0.00%	0.00%	0.18 ± 0.08 (0)	96.00%	0.00%	4.00%	0.00%	0.00%	0.08 ± 0.06 (0)	0.436
Incontinence	74.00%	20.00%	6.00%	0.00%	0.00%	0.32 ± 0.08 (0)	84.00%	10.00%	2.00%	4.00%	0.00%	0.26 ± 0.10 (0)	0.305
Sexual symptom						2.58 ± 0.17 (3)						1.62 ± 0.20 (1)	**< 0.005**
Decreased libido	8.00%	8.00%	32.00%	22.00%	30.00%	2.58 ± 0.17 (3)	77.80%	24.00%	20.00%	14.00%	14.00%	1.62 ± 0.20 (1)	**<0.005**
GSM						5.84 ± 0.49 (5)						3.20 ± 0.41 (2.5)	**< 0.005**

*P-value in bold indicates statistical difference is significant.*

### Sequencing Data, Microbial Distribution, and Abundance

In this work, a total of 3,965,265 sequence reads (range 30,214–72,367 reads per sample) were acquired after quality control, with an average length of 444 bp per sample. Then high-quality sequences were classified into 27 phyla, 52 classes, 103 orders, 176 families, and 356 genera through bioinformatics processing.

The alpha diversity indices differed significantly between the non-treated group and MHT group as shown in Table 3. Shannon diversity index, Ace richness index, and Chao estimator were greater in the non-treated samples, whereas Simpson diversity index was lower in the non-treated samples relative to the MHT samples (*P* < 0.005). These results reflected a higher microbial diversity of vaginal environment in the non-treated women than in the MHT-treated women. However, there was no significant difference in Good’s coverage value between two groups, and the value for each group was 1.00, suggesting that the sequencing depth was sufficient to saturate the taxa.

**TABLE 3:** Alpha diversity indices for vaginal microbiota.

Alpha diversity indices	Non-treated group (*n* = 50)			MHT group (*n* = 50)			*P*-values
	Median	Q1	Q3	Median	Q1	Q3	
Shannon	0.99	0.59	1.46	0.02	0.01	0.52	**< 0.005**
Simpson	0.48	0.30	0.70	1.00	0.70	1.00	**< 0.005**
Ace	51.53	24.43	111.52	30.58	12.54	63.07	**< 0.005**
Chao	35.13	21.75	107.00	22.10	10.75	30.44	**< 0.005**
Coverage	1.00	1.00	1.00	1.00	1.00	1.00	0.109

*P-values were obtained from Mann-Whitney U-test. Bold value means there exists significant statistical difference.*

**FIGURE 2:**
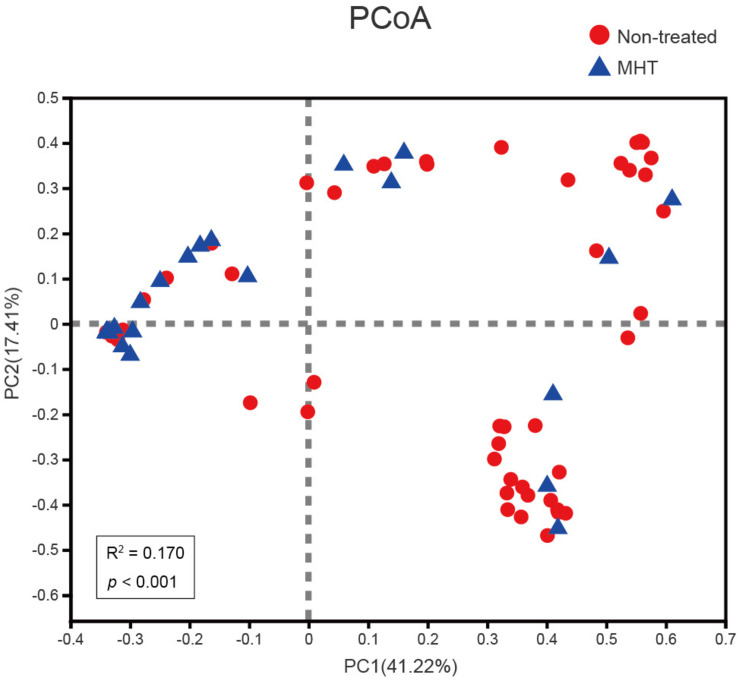
Comparing samples distributions belonging to the non-treated (red) or MHT (blue) group by using principal coordinate analysis (PCoA) on the Bray–Curtis dissimilarity. Each sample is shown as a dot.

To examine how the vaginal microbiota varied between these two groups, beta diversity analysis was performed by PCoA on the Bray–Curtis dissimilarity. As illustrated in Figure 2, the community structure of MHT-treated women was mostly separated from non-treated women (Adonis *P* < 0.001), with an *R*^2^-value (0.170) suggestive of this significant differentiation.

**FIGURE 3:**
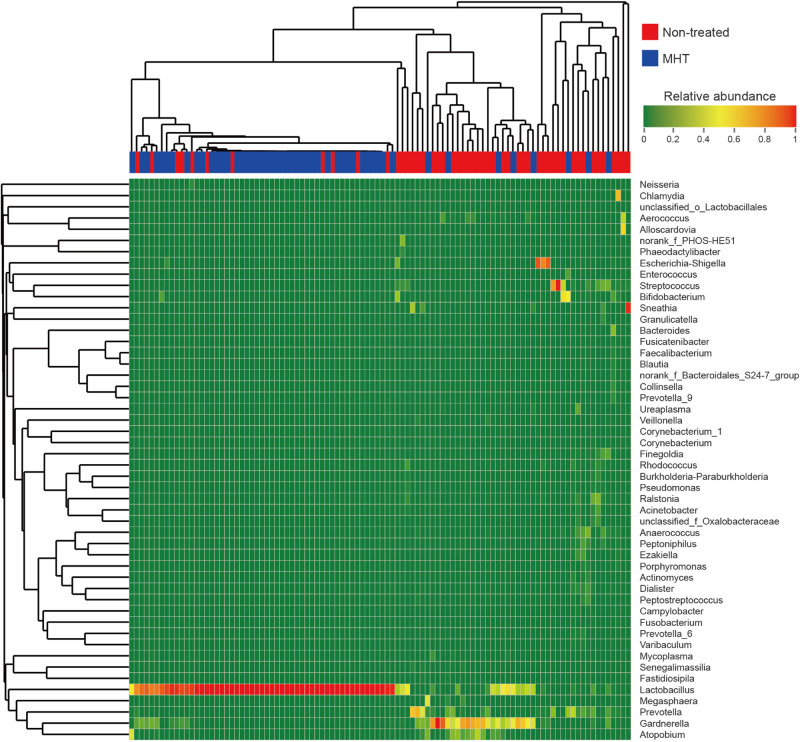
Heatmap analysis of relative abundance of microbial taxa found in all vaginal samples at the genus level (top 50). Each sample is shown as vertical bar, and different color of each cell represents different relative abundance: the low abundance and high abundance are highlighted by green and red, respectively. The horizontal bar on the top indicates the non-treated (red) or MHT (blue) group; dendrograms reflect hierarchical clustering on Bray–Curtis dissimilarity (columns) or Euclidean distance (rows) using average linkage.

For more details on microbial community at the genus level, we analyzed the relative abundance of the top 50 classified genera in all samples, which was demonstrated as a hierarchically clustered heatmap (Figure 3). Table 4 lists the most abundant genera in two groups and included only the genera with abundance proportions greater than 0.005 in each sample; those failing to meet the condition were expressed as “others.” According to Figure 3, samples within the same group were more interrelated. Furthermore, microbial community profiles from the two groups were markedly different (Figure 3 and Table 4), which was also verified by PCoA results. *Lactobacillus* was the most representative bacterial genus in both groups, but was dominant only in the MHT group (*Lactobacillus* proportion of 29.1% in the non-treated samples vs. 83.1% in the MHT samples). In the non-treated group, *Gardnerella*, *Prevotella*, *Streptococcus*, *Escherichia-Shigella*, *Atopobium*, *Sneathia*, *Bifidobacterium*, etc., made great contributions, which was demonstrated by both data and graphics.

**TABLE 4:** Proportion of most abundant genera in vaginal microbiota of different groups.

Genus	Abundance proportion in non-treated group			Abundance proportion in MHT group		
	Mean	Max	Min	Mean	Max	Min
*Lactobacillus*	29.1%	100.0%	0.0%	83.1%	100.0%	0.0%
*Gardnerella*	22.7%	99.9%	0.0%	6.4%	55.5%	0.0%
*Prevotella*	7.4%	63.5%	0.0%	1.7%	31.4%	0.0%
*Streptococcus*	5.8%	97.4%	0.0%	0.7%	21.4%	0.0%
*Escherichia-Shigella*	5.1%	81.4%	0.0%	0.2%	9.2%	0.0%
*Atopobium*	4.4%	37.0%	0.0%	2.1%	46.0%	0.0%
*Sneathia*	3.1%	92.5%	0.0%	0.1%	6.6%	0.0%
*Bifidobacterium*	2.1%	54.1%	0.0%	1.4%	52.0%	0.0%
*Aerococcus*	1.8%	38.4%	0.0%	0.1%	1.9%	0.0%
*Ralstonia*	1.5%	22.2%	0.0%	0.1%	1.3%	0.0%
*Anaerococcus*	1.3%	15.1%	0.0%	0.6%	23.6%	0.0%
*Chlamydia*	1.2%	58.6%	0.0%	0.0%	0.0%	0.0%
*Alloscardovia*	1.1%	56.1%	0.0%	0.0%	0.2%	0.0%
*Rhodococcus*	0.8%	11.3%	0.0%	0.0%	0.8%	0.0%
*Peptoniphilus*	0.7%	13.2%	0.0%	0.2%	7.5%	0.0%
*Finegoldia*	0.6%	15.1%	0.0%	0.4%	17.6%	0.0%
*Acinetobacter*	0.6%	14.1%	0.0%	0.0%	0.1%	0.0%
*Ezakiella*	0.6%	12.3%	0.0%	0.0%	2.1%	0.0%
*Dialister*	0.5%	5.4%	0.0%	0.3%	11.6%	0.0%
*Ureaplasma*	0.5%	18.0%	0.0%	0.2%	4.5%	0.0%
*Megasphaera*	0.2%	9.8%	0.0%	1.1%	46.4%	0.0%
Others	8.7%	72.9%	0.0%	1.4%	30.3%	0.0%

*Others represent those genera with abundance proportion less than 0.005 in each sample. Mean, the average proportion of all samples in one group; Min, minimal proportion of a sample in one group; Max, maximal proportion of a sample in one group.*

### Significant Differences in Microbial Communities

**FIGURE 4:**
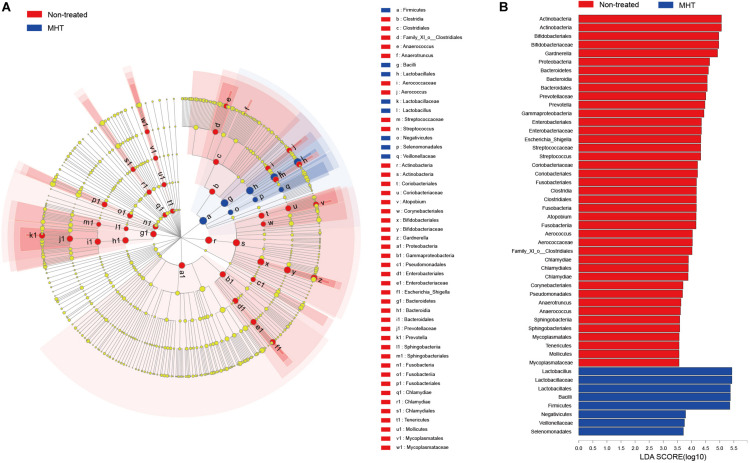
Linear discriminant analysis (LDA) effect size (LEfSe) analysis for comparing microbial variations at the genus level between the non-treated (red) and MHT (blue) group. **(A)** LEfSE cladogram of microbial taxa in different group (*P* < 0.05). **(B)** LDA score identifying effect size of difference between groups with a threshold value of 3.5, a higher score represents a greater contribution to the observed intergroup difference, indicating this taxon is a more important biomarker.

Subsequently, biomarker analysis was conducted to identify the special microbial taxa with significant abundance differences between non-treated and MHT samples using the LEfSe modeling. As seen in Figure 4, there were 49 bacterial clades presenting significant intergroup differences with a LDA threshold of 3.5, and most bacteria were enriched in non-treated samples. Focusing on the genus level, we found that there were as many as eight significantly different genera in the non-treated group, namely, *Gardnerella*, *Prevotella*, *Escherichia-Shigella*, *Streptococcus*, *Atopobium*, *Aerococcus*, *Anaerotruncus*, and *Anaerococcus* (all *P* < 0.05). In contrast, only *Lactobacillus* manifested the abundance advantage in the MHT group (*P* < 0.05).

### Multivariate Analysis

**FIGURE 5:**
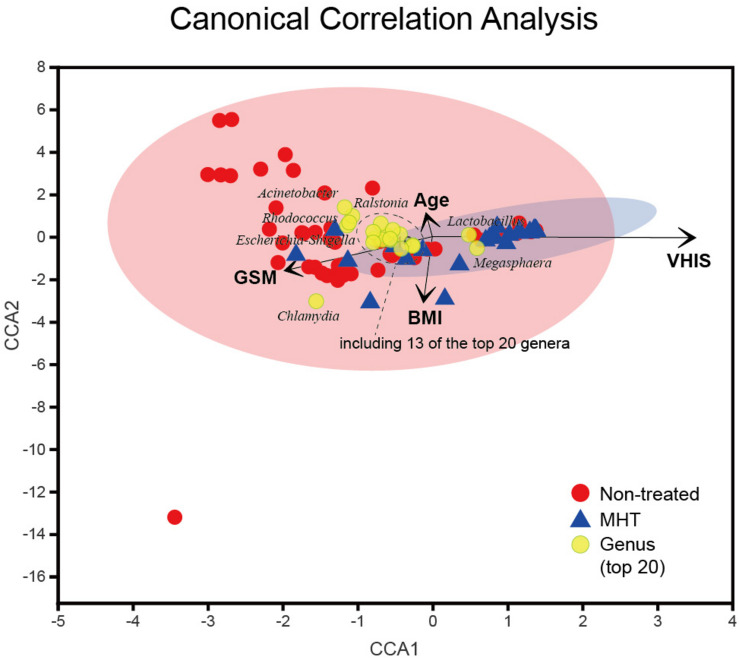
Canonical correlation analysis (CCA) of the above vaginal microbiota and clinical characteristics. Red and blue points represent non-treated and MHT samples, respectively, the 95% confidence intervals of samples’ distribution are visualized by ellipses, and the top 20 microbial genera are colored with yellow. Clinical characteristic variables (age, BMI, VHIS, GSM) are indicated by vectors in black.

Finally, the CCA was applied to reveal the potentially multivariate correlation of clinical characteristics and primary microbial community (Figure 5). In the CCA, the length of the vector is used to interpret this correlation (a longer vector means a greater correlation with bacterial distribution), and the angle between vectors could reflect associations of clinical variables (a positive association is plotted as an acute angle and a negative association as an obtuse angle). Additionally, a greater distance between samples indicates a stronger dissimilarity in microbial structure. According to Figure 5, we found that the dissimilarity between samples in the non-treated group was larger than that in the MHT group, this phenomenon was consistent with PCoA results. Moreover, the distribution of non-treated samples was generally in accordance with the GSM vector, whereas MHT samples were mostly distributed in the direction of the VHIS vector, which was similar to the comparison of the severity of GSM between these two groups. The first axis had a significantly positive correlation with VHIS (*P* < 0.001), as well as a negative correlation with GSM (*P* < 0.005); it was obvious that these two variables had an obtuse relationship. *Lactobacillus* was also associated with the first axis; similarly, it corrected with VHIS positively (*r* = 0.626, *P* < 0.001) and with GSM negatively (*r* = −0.347, *P* < 0.001). As for GSM, other vaginal genera had some connections with it as well, including *Chlamydia* (*r* = 0.277, *P* < 0.01), *Streptococcus* (*r* = 0.270, *P* < 0.01), *Enterococcus* (*r* = 0.236, *P* < 0.05), *Corynebacterium* (*r* = 0.234, *P* < 0.05), *Porphyromonas* (*r* = 0.223, *P* < 0.05), and *Escherichia-Shigella* (*r* = 0.199, *P* < 0.05), all of which increased with the GSM severity score. Additionally, the second axis only presented a significantly inverse relationship with BMI (*P* < 0.001), but age could not affect the microbial community distribution in our study (*P* > 0.05).

## Discussion

In the current study, we performed a cross-sectional survey to determine a vaginal microbial restoration and GSM-related symptom improvement with MHT application based on 16S rRNA gene sequencing and to demonstrate the salient relevance among them. Taking into account the possible impact of different MHT options on the outcome, we chose tibolone as the only unified treatment. The foremost reason is that the vast majority of women treated with tibolone had no menstrual period; in this way, effects of menstruation on vaginal flora could be eliminated. What needs to be explained was that in our study cohort, patients received tibolone treatment due to multiple systematic symptoms rather than genitourinary symptoms alone, in strict accordance with the medicine guidelines (Pinkerton, 2020). Tibolone, a synthetic steroid with estrogenic, progestogenic, and androgenic properties, has long been used worldwide for menopausal symptoms (Formoso et al., 2016). Obviously, we proved that women receiving tibolone had significantly milder GSM symptoms than non-treated women, especially for vulvovaginal dryness and burning, as well as decreased libido, which was similar to previously reported studies (Kenemans and Speroff, 2005). However, there was no significant distinction in vulvovaginal itching and all urological symptoms between the two populations. The underlying basis for these findings is presumably the medicinal property. Tibolone is rapidly metabolized into three metabolites that play diverse roles in target tissues in the body through binding with estrogen, progesterone, or androgen receptors and modulating enzymatic activity (Kloosterboer, 2004), which explains why the levels of serum E_2_ and FSH were steady in all study participants. Tibolone itself and its Δ4 isomer have slight androgenic effects contributing to the increases in free testosterone and consequently to improving sexual desire (Laan et al., 2001). The female reproductive and lower urinary tracts share the same embryonic origin—urogenital sinus; furthermore, estrogen, and progesterone receptors have been identified in both tissues (Tincello et al., 2009). In theory, it could be anticipated that hormone therapy would help to ameliorate both genital and urinary symptoms, whereas the present researches still remain controversial about the effect of systemic estrogen on urinary symptoms (Robinson and Cardozo, 2011). We supposed that the urinary tract symptoms were mainly mild in the cohort, which to some extent may explain the lack of significant remission of symptoms after tibolone. Hence further clinical surveys and basic experiments should be carried out to clarify the management of postmenopausal lower urinary tract symptoms.

Herein, we demonstrated the vaginal microflora of women with MHT differed from that of non-treated women. First, the differences in all alpha diversity indices between groups implied a higher microbial diversity in samples of non-treated women, which suggested that there might be no definitely preponderant bacteria in these samples. Then, the analysis results revealed distinct vaginal microbial signatures in each group. *Lactobacillus* accounted for the highest proportion in both of them, but it was only dominant in the vaginal community of women with MHT. We also found it correlated positively with a healthier vaginal condition as well as negatively with more severe GSM, corresponding to the findings of previous studies (Muhleisen and Herbst-Kralovetz, 2016; Mitchell et al., 2018; Gliniewicz et al., 2019). However, it is pertinent to note that the non-treated vaginal microflora significantly included several anaerobic bacteria, namely, *Gardnerella*, *Prevotella*, *Escherichia-Shigella*, *Streptococcus*, *Atopobium*, *Aerococcus*, *Anaerotruncus*, and *Anaerococcus*. Interestingly, *Prevotella* has been shown to promote the growth of *Gardnerella* by producing polyamines during metabolism, in the same way, the existence of biofilm in *Gardnerella* could stimulate the growth of *Prevotella* (Randis and Ratner, 2019). Additionally, not only *Gardnerella* and *Prevotella* but also *Streptococcus* and *Atopobium* were strongly associated with bacterial vaginosis (Onderdonk et al., 2016). Numerous studies have affirmed that *Escherichia coli*, as the primary pathogen of urinary tract infections, could adhere to or even invade into vaginal epithelial cells prior to inducing an ascending infection (Brannon et al., 2020). Likewise, a few particular species of *Aerococcus* were also believed to involve in urinary tract infections (Rasmussen, 2016). One previous research detected that *Anaerotruncus* in the female reproductive tract might contribute to the development of endometrial cancer (Walther-António et al., 2016). Moreover, *Anaerococcus* was reported to be correlated with cervical intraepithelial neoplasia disease progression (Mitra et al., 2015). Taken together, these findings hint that ecological interactions are meaningful for vaginal environment and reproductive health; however, the potential interactions are far from definitive.

Our work specifically addressed relationships between the severity of GSM and vaginal microbiota, and except for a strikingly negative correlation between *Lactobacillus* and GSM, we demonstrated that other taxa correlated with GSM positively, including *Chlamydia* and *Streptococcus*, with high correlation coefficients. It has been suggested that a distinct bacterial community state characterized by *Streptococcus* was associated with vulvovaginal atrophy (Brotman et al., 2018), and our results were consistent with this finding. Moreover, it is well known that *Chlamydia* infection could result in acute and chronic reproductive disorders. Nevertheless, a few cross-sectional studies found that women in menopause were not susceptible to *Chlamydia* (Jackson et al., 2015; Stemmer et al., 2018). Notably, there was probably a complex mutual mechanism underlying the definite association between *Chlamydia* and more severe symptoms of GSM, as shown in our findings. Infection with *Chlamydia* in the female genital tract could drive increased expression of TLR2 and TLR4 accompanied by upregulation of cytokines, such as tumor necrosis factor α, interleukin 1α (IL-1α), and IL-6; these cytokines together trigger the inflammatory response during which tissue damage occurs (Agrawal et al., 2009). Unfortunately, our study did not involve immune-relevant research, so there was no way to directly provide an explicit conclusion of the interaction mechanism between vaginal bacteria and the local immune response. This aspect was one of limitations in the present study. However, based on the available evidence, we may speculate that the composition and stability of the vaginal microbiota play an essential part in determining the mucosal immune response and susceptibility to infection. Other limitations included that sample sizes were relatively small and participants included in this study were only from outpatients in one clinical center, which might lead to the issue that our results only partly reflected the situation of menopausal women in Shanghai, China. In this regard, additional studies with larger sample sizes and multicenter are required to further investigate the latent microbes associated with GSM and the microbiological mechanism in the occurrence and development of GSM.

In conclusion, GSM emerges with a decline in circulating estrogen levels, which may worsen gradually without intervention. Understanding the microbiological effect of MHT on GSM could help to improve novel strategies for GSM treatment and prevent discomfort. Our study demonstrated that *Lactobacillus* was distinctly abundant in the vagina and that symptoms of GSM were notably alleviated after regular hormone therapy. Furthermore, several special anaerobic bacteria were found to be significantly enriched in menopausal women without any treatment, and these bacteria were bound up with gynecological and reproductive diseases. Women infected with *Chlamydia* or *Streptococcus* in the vagina may be prone to suffer from GSM, which is necessary to be taken into account by medical staff. These findings are of great importance to clinical practice for the management of GSM, although the microbiological mechanism in the progression of GSM remains to be explored and perfected further.

## Data Availability Statement

The datasets presented in this study have been deposited in an online repository. The name of the repository and accession number can be found below: https://www.ncbi.nlm.nih.gov/, PRJNA669119.

## Ethics Statement

The studies involving human participants were reviewed and approved by the Ethics Committee of Shanghai Sixth People’s Hospital. The participants provided their written informed consent to participate in this study. Written informed consent was obtained from the individuals for the publication of any potentially identifiable images or data included in this article.

## Author Contributions

MT conceived, directed and coordinated all aspects of this study. LG, WH, SJ, YanwZ, and PL participated in sample collection, the data collation, and result analysis. YiZ, YangZ, and JH contributed to DNA extraction and PCR amplification from samples. LG and MT prepared and edited the manuscript. All authors contributed to the article and approved the submitted version.

## Conflict of Interest

The authors declare that the research was conducted in the absence of any commercial or financial relationships that could be construed as a potential conflict of interest.

## Footnotes

1 https://www.arb-silva.de/

2 http://rdp.cme.msu.edu/

3 http://huttenhower.sph.harvard.edu/galaxy/
